# Boerhaave’s Syndrome: An Unusual Geriatric Presentation

**DOI:** 10.7759/cureus.46212

**Published:** 2023-09-29

**Authors:** Cristiana Canelas Mendes, Leila Duarte, João Madeira Lopes

**Affiliations:** 1 Internal Medicine, Santa Maria Hospital, Centro Hospitalar Universitário Lisboa Norte (North Lisbon University Hospital Center), Lisbon, PRT

**Keywords:** endoscopic oesophageal prosthesis, haemorrhagic shock, empyema, pneumomediastinum, surgical oesophageal prosthesis, spontaneous oesophageal perforation, boerhaave's syndrome

## Abstract

Boerhaave's syndrome (BS) is a non-iatrogenic spontaneous esophageal perforation that, if not appropriately recognized and managed, can cause localized infections such as mediastinitis, pneumonia, and empyema, as well as systemic infections with significant morbidity and mortality rates. An autonomous 83-year-old male presented to the emergency department with a three-day history of behavioral changes. Three days earlier, the patient had a self-limited episode of cough, nonspecific thoracalgia, palpitations, prostration, and pallor. On physical examination, he was alert but had temporal disorientation, hypoxemia, and pulmonary auscultation with abolished breath sounds in the middle third of the left chest. Laboratory tests showed hypoxemia, elevated C-reactive protein (28.2 mg/dL), and D-dimer (3.28 µg/mL). A chest X-ray revealed periaortic small bubbles, left atelectasis, and left pleural effusion. Computed tomographic angiography of the chest showed infra-carinal esophageal rupture, small bubbles of the anterior pneumomediastinum, and a loculated left pleural empyema. Mediastinitis and empyema due to BS were assumed. He underwent left thoracic drainage, broad-spectrum antibiotics, and the placement of a surgical esophageal prosthesis. He was discharged after 48 days.

The condition known as BS is frequently misdiagnosed, mostly as a result of the lack of a preexisting pathological background and the wide array of potential symptoms that may manifest. The diagnosis in this particular case was rendered particularly complex due to the combination of an unusual presentation and a delayed seeking of medical attention. Against all expectations, our patient was successfully treated.

## Introduction

Esophageal perforation and rupture (EPR) is a rare and potentially life-threatening disease if not treated properly. Both traumatic and non-traumatic factors can contribute to EPR. Non-traumatic factors are classified as iatrogenic or non-iatrogenic. Iatrogenic complications occur during or after endoscopic procedures. Boerhaave's syndrome (BS) is a non-iatrogenic spontaneous esophageal perforation that is even rarer, accounting for 15% of all EPR occurrences [1-3].

The majority of spontaneous lesions occur in men between the ages of 50 and 70. They can only affect part of the esophageal wall (as in Mallory-Weiss syndrome, which occurs at the gastro-esophageal junction) or cause a full-thickness organ rupture, which leads to BS [4]. The term "spontaneous" does not imply the absence of a triggering element but rather that the rupture is not the result of direct trauma (often produced by instrumentation or a foreign body). Vomiting is the most prevalent triggering event related to BS, but other factors such as straining, lifting, or even laughing have been described [1]. If it is not appropriately recognized and managed, it can cause localized infections such as mediastinitis, pneumonia, and empyema, as well as systemic infections with significant morbidity and mortality rates [1-3].

## Case presentation

**Figure 1:**
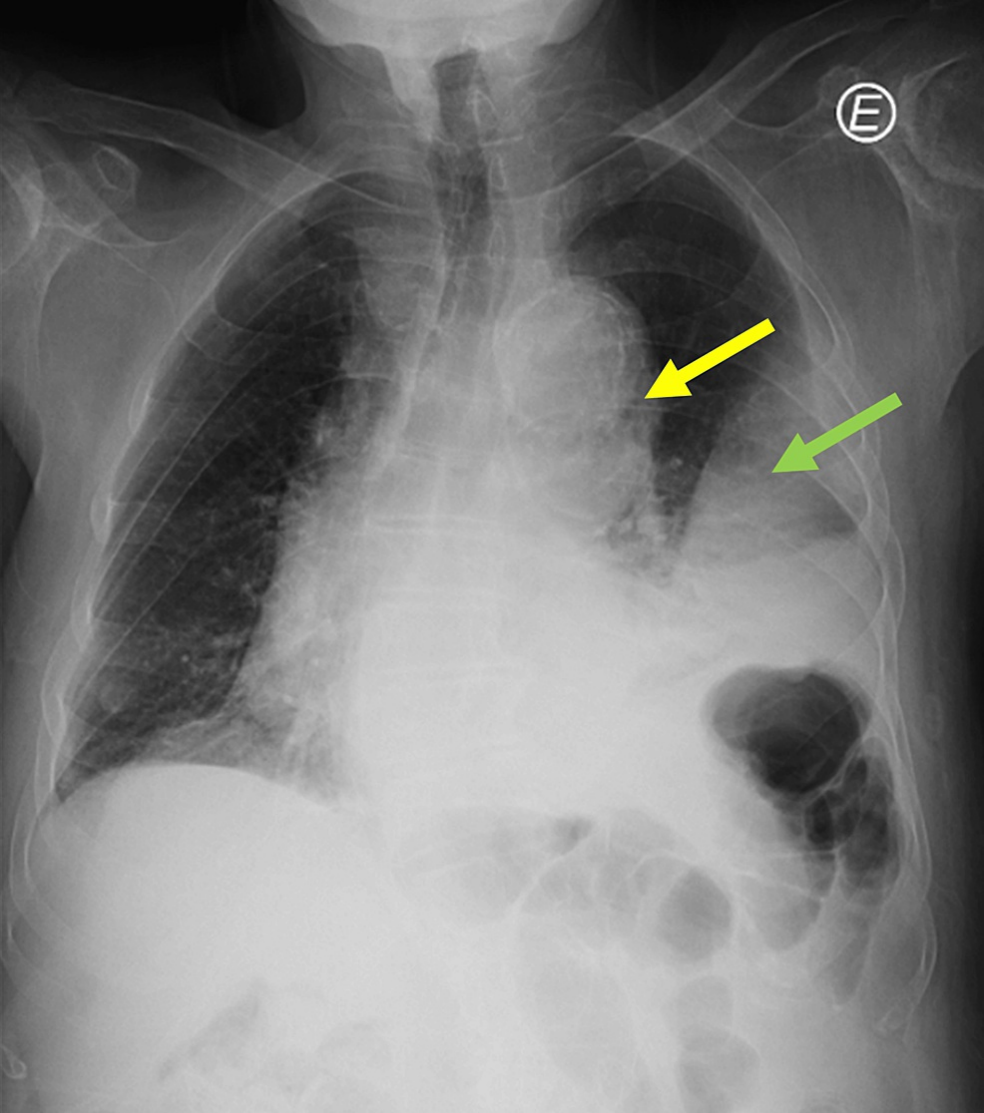
Chest X-ray with periaortic small air bubbles (pneumomediastinum) marked by the yellow arrow, atelectasis, and left pleural effusion (green arrow)

An autonomous 83-year-old male (Eastern Cooperative Oncology Group (ECOG) performance status scale 2) with a prior history of arterial hypertension, mild to moderate aortic valve stenosis, and a thyroid nodule with normal thyroid studies presented at the emergency department with a three-day history of behavioral changes denied trauma or previous surgical history. Three days earlier, he had a self-limited episode of cough, nonspecific thoracalgia, palpitations, prostration, and pallor. On physical examination, he was alert but had temporal disorientation. His saturation of peripheral oxygen (SpO2) in room air was 92%, and pulmonary auscultation showed an absence of breath sounds in the middle third of his left chest. Laboratory tests revealed hypoxemic respiratory failure: pH 7.49 (7.35-7.45), partial pressure of carbon dioxide (pCO2) 32.8 mmHg (35-45), partial pressure of oxygen (pO2) 57.6 (75-100), bicarbonate (HCO3) 26.2 mmol/L, elevated C-reactive protein (CRP) 28.2 mg/dL (<0.5), and D-dimer 3.28 g/mL (0-0.5). The chest X-ray revealed pneumomediastinum with periaortic small air bubbles, left atelectasis, and left pleural effusion (Figure 1).

**Figure 2:**
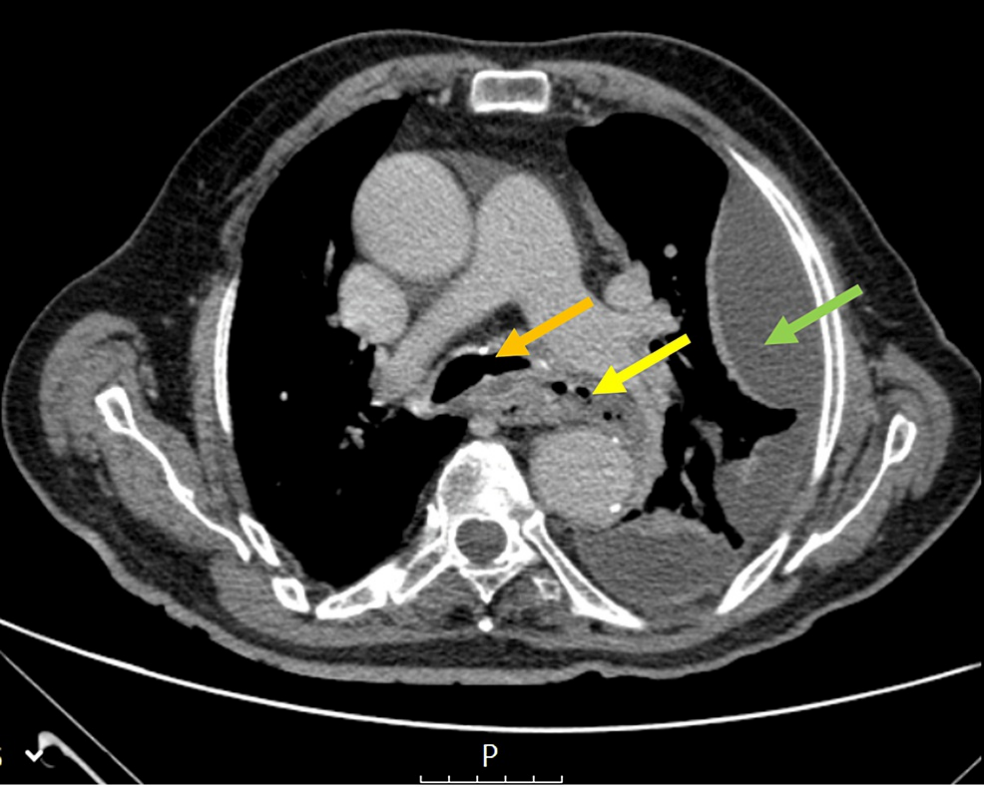
Computed tomographic angiography Yellow arrow: Infra-carinal esophageal rupture with pneumomediastinum, Green arrow: Left pleural empyema, Orange arrow: Carina

A computed tomographic angiography of the chest showed an infra-carinal esophageal rupture, small bubbles of anterior pneumomediastinum, and a loculated left pleural empyema (Figure 2).

Mediastinitis and empyema due to BS were assumed. Piperacillin/tazobactam, vancomycin, metronidazole, and fluconazole were started, and left chest tube drainage was performed. A surgical esophageal prosthesis was placed almost 72 hours after symptom onset. The microbiological culture of blood, urine, and pleural fluid was negative. On the seventh day of the critical care stay, the prosthesis migrated, and the patient went into hemorrhagic shock. Repositioning was performed by endoscopy, and the patient was discharged after 48 days of full recovery.

## Discussion

Mackler's triad involves vomiting, chest pain, and subcutaneous emphysema, which is the classic clinical presentation of BS. Nevertheless, this triad is only present in 10% of cases. The most common symptom is chest discomfort, which is reported by 70% of patients. Other symptoms included dyspnea, dysphagia, tachycardia, fever, tachypnea, epigastric discomfort, and, in rare cases, hematemesis or other evidence of gastrointestinal bleeding, such as melenas [1-4].

A sudden rise in intraesophageal pressure, when combined with negative intrathoracic pressure, produces a traction force that causes BS. This occurs, for example, when there is violent vomiting and/or retching. More than 70% of perforations are thoracic, involving the posterolateral section of the distal esophagus, as in our case, but they can also occur in other parts of the esophagus, such as the intra-abdominal or cervical esophagus [5,6].

Atypical presentations can make the diagnosis even more challenging, increasing morbidity and mortality [7,8]. Although the exact morbidity of this syndrome is unknown, it is considered high [5]. The importance of early clinical suspicion cannot be overstated. Although modern diagnostic techniques have significantly contributed to various areas of contemporary clinical practice, diagnosing esophageal perforations remains challenging. This difficulty can lead to significant delays in treatment, ultimately resulting in decreased survival rates, even in high-volume medical centers [7,9,10].

In cases where there is a clinical suspicion for BS, medical professionals may opt to conduct chest radiography, esophagography, and a CT scan to definitively establish the diagnosis. In the context of CT findings, the peri-esophageal air collections observed at the supradiaphragmatic level are identified as the most valuable indicators in patients with spontaneous perforation. Chest radiography holds significant value within the emergency department, although its diagnostic capabilities for patients with BS are limited in specificity. According to existing research, the prevailing observations in radiography of individuals with spontaneous esophageal perforation mostly involve the presence of pleural effusions accompanied by infiltrates that exhibit a left-side predominance. Subsequently, atelectasis is also frequently observed [9,11].

Computed tomography imaging is an essential diagnostic tool for patients suspected of having BS, as it can provide crucial evidence of periesophageal air tracks that indicate esophageal perforation. Therefore, it is recommended that CT scans be included in the diagnostic evaluation of patients when BS is considered a potential diagnosis [9].

There is a common belief that the mortality rate of this condition, which can be as high as 40% to 60% for patients treated after 48 hours, is due to a delayed diagnosis and that the prognosis improves when treatment starts within the first 24 hours. However, a study of 79 patients showed that a delayed diagnosis did not have a statistically significant effect. Another finding indicated that there was an elevated mortality risk associated with higher values of both EPR size and the percentage of segmented neutrophils in the WBC count [2].

The management of EPR is contingent upon various factors, including the etiology, anatomical site, dimensions, concomitant manifestation of symptoms, and patient location. The management of BS typically involves either primary repair of the injured location or endoscopic insertion of a self-expanded stent, particularly when surgical settings are not favorable [2,3]. In 2013, a European retrospective study of 38 patients found that there were no clear advantages of endoscopic therapy over surgery in terms of morbidity and length of stay in the intensive care unit and that it was associated with frequent treatment failure eventually requiring surgical intervention [12]. Endoscopic treatment is often chosen for older patients since it is less invasive. Taking into account the good general condition and performance status of our patient, the approach chosen was surgical. The prosthesis eventually migrated due to the laceration of the mucosa, but it was possible to repair it endoscopically, and the patient survived.

## Conclusions

The condition known as BS is frequently misdiagnosed, mostly as a result of the lack of a preexisting pathological background and the wide array of potential symptoms that may manifest. The diagnosis in this particular case was rendered particularly complex due to the combination of an unusual presentation and a delayed seeking of medical attention. Against all expectations, our patient was successfully treated.
